# Recent loss of closed forests is associated with Ebola virus disease outbreaks

**DOI:** 10.1038/s41598-017-14727-9

**Published:** 2017-10-30

**Authors:** Jesús Olivero, Julia E. Fa, Raimundo Real, Ana L. Márquez, Miguel A. Farfán, J. Mario Vargas, David Gaveau, Mohammad A. Salim, Douglas Park, Jamison Suter, Shona King, Siv Aina Leendertz, Douglas Sheil, Robert Nasi

**Affiliations:** 1 Grupo de Biogeografía, Diversidad y Conservación, Departamento de Biología Animal, Fac. Ciencias, Universidad de Málaga, 29071, Málaga, Spain; 2 Division of Biology and Conservation Ecology, School of Science and the Environment, Manchester Metropolitan University, Manchester, M15 6BH UK; 3 Center for International Forestry Research (CIFOR), Jalan Cifor Rawajaha, Situ Gede, Bogor Barat, Kota Bogor, Jawa Barat 16115 Indonesia; 4 ERM Foundation, Exchequer Court, 33 St Mary Axe, London, EC3A 8AA UK; 5 The Environment Foundation for Africa, 16 Main Peninsula Road, Sussex, Freetown, Sierra Leone; 6 Epidemiology of Highly Infectious Microorganisms, Robert Koch Institute, Seestrasse 10, 13353 Berlin, Germany; 7 Department of Infectious Disease Epidemiology, Robert Koch Institute, Seestrasse 10, 13353 Berlin, Germany; 8 Faculty of Environmental Sciences and Natural Resource Management, Norwegian University of Life Sciences, P.O. Box 5003, NO-1432 Ås, Norway

**Keywords:** Ecological epidemiology, Biogeography

## Abstract

Ebola virus disease (EVD) is a contagious, severe and often lethal form of hemorrhagic fever in humans. The association of EVD outbreaks with forest clearance has been suggested previously but many aspects remained uncharacterized. We used remote sensing techniques to investigate the association between deforestation in time and space, with EVD outbreaks in Central and West Africa. Favorability modeling, centered on 27 EVD outbreak sites and 280 comparable control sites, revealed that outbreaks located along the limits of the rainforest biome were significantly associated with forest losses within the previous 2 years. This association was strongest for closed forests (>83%), both intact and disturbed, of a range of tree heights (5–>19 m). Our results suggest that the increased probability of an EVD outbreak occurring in a site is linked to recent deforestation events, and that preventing the loss of forests could reduce the likelihood of future outbreaks.

## Introduction

Ebola virus disease (EVD) is a zoonosis that causes severe and often fatal haemorrhagic fever in humans^1^. EVD was first identified in Africa in 1976^2^ and since then is estimated to have killed over 13,000 people^3^. Due to its associated high mortality and potential for contagion, EVD is viewed as a global threat^4^. EVD is propagated by a group of filovirus species of the genus *Ebolavirus* (hereafter Ebola virus)^1^, but despite advances in understanding this zoonotic disease, the factors that trigger and maintain outbreaks remain elusive^5^. Such uncertainties impede the more accurate and effective prediction of outbreaks that would facilitate improved response or prevention^6^. Human activities may have promoted direct or indirect contact between humans and an animal reservoir of the virus^7^. Some suggest that the loss of forest can facilitate the spread of the disease to non-forest areas^8,9^. The mechanism, although unknown, likely results from more frequent contact between infected wild animals and humans. The latest outbreak in Guinea has been linked to contact with a bat colony, an event that some have linked to forest loss^10,11^. However, the enabling role of forest loss in Ebola outbreaks seems hard to reconcile with the upper Guinea forests having been a dynamic mosaic of forest, savannah, and farmland for centuries, and that people in this region have been sympatric with bats, and other forest wildlife, throughout this history^12^. More generally, humans^13^ and great apes^14^ have lived in close proximity to bats for millennia, thus it may be simplistic to claim that forest loss was sufficient to cause the emergence of EVD and its repeated outbreaks.

Initial suggestions that deforestation increases zoonotic EVD outbreaks result from observations in seven West African EVD outbreak sites that revealed greater forest fragmentation in these locations than in their surroundings^15^. Quantitative analysis of the nexus between deforestation and the emergence of Ebola virus disease has been recently undertaken by Rulli *et al*.^16^. Although this study showed that EVD outbreaks occurred mostly in hotspots of forest fragmentation, the spatio-temporal dynamics of this relationship was not considered. Here, using forest change remote sensing data^17^ and modeling we investigate: (1) the spatio-temporal association between forest types and forest changes and the possibility of an outbreak, and (2) whether this association can be extended to the whole distribution range of the Ebola virus in West and Central Africa^18^.

In this study, our original hypothesis was that deforestation leads to increased contacts between humans and hosts or vulnerable mammals and thus leads to zoonotic outbreaks of EVD. The underlying assumption is that zoonotic transmission would be more probable either because hunters would travel further (or therefore increase possible contacts) or because in fragmented forests there can be an increased density of bats and other potential reservoirs of Ebola virus^7–9^. In order to test these relationships, we investigated vegetation-cover changes within human populated areas where Ebola outbreaks have occurred and compared these to otherwise comparable localities where there have not been outbreaks. Out of the 40 EVD outbreaks reported since 1976 we were able to focus on 27 sites where index cases (the first patient that indicates the existence of an EVD outbreak) were identified (Table S1) and for which large-scale deforestation data were available for the period 2001–2014^17^. We used spatial distribution modeling based on the Favorability Function^19^ to develop spatio-temporal distribution models using a set of predictor variables (Table S2) to discriminate between EVD outbreak locations and 280 control spatio-temporal locations (randomly selected sites containing human settlements but no recorded EVD outbreaks) (Figure S1). We assessed annual forest loss and fragmentation within a 20-km radius buffer around each outbreak and non-outbreak site (See Methods); the buffer radius reflecting the distance hunters typically range from their villages^20,21^ and thus defines the area where we assume zoonotic disease transmission could occur.

## Results and Discussion

**Figure 1:**
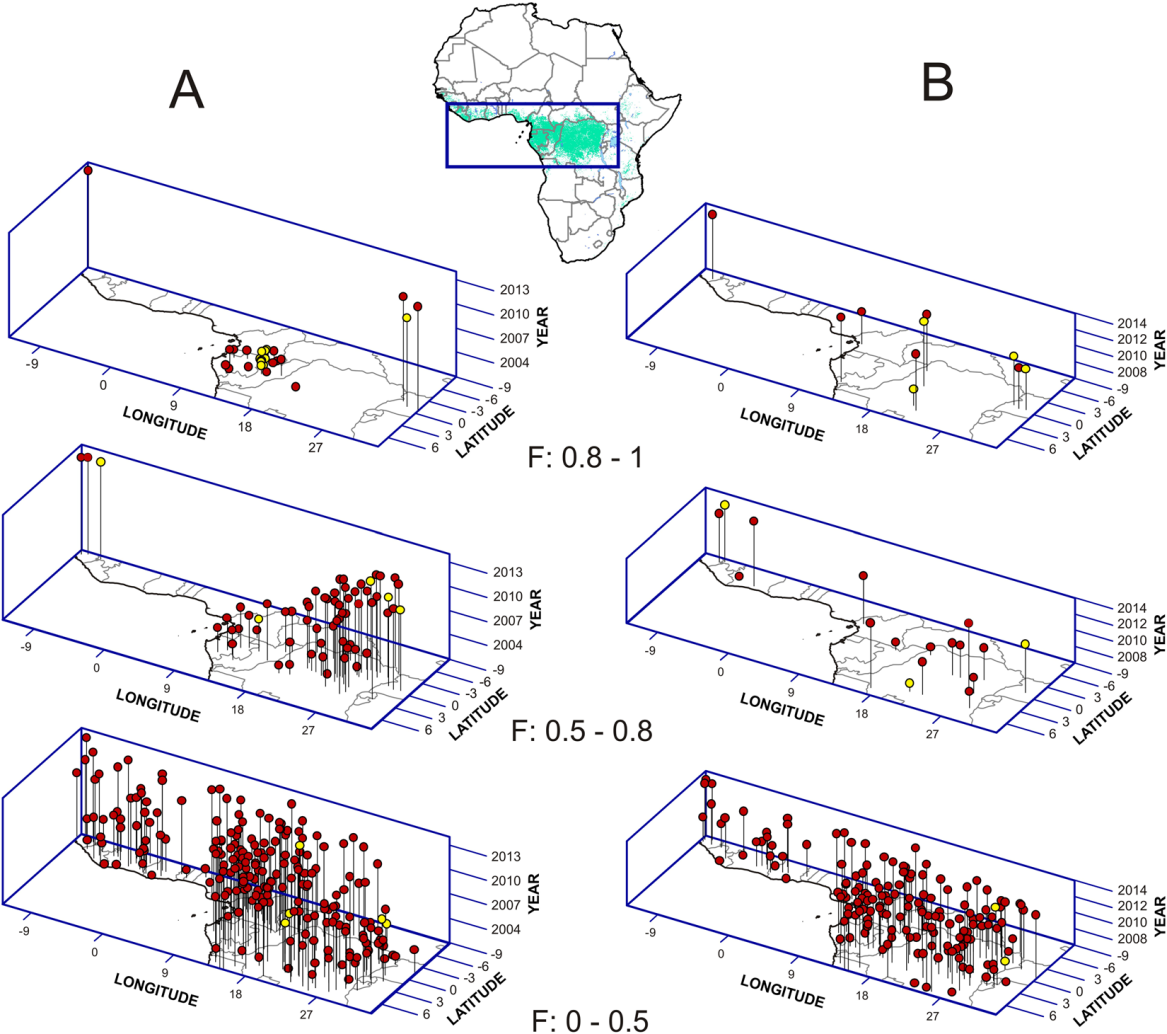
3D representations of two models of favorability (F) for the occurrence of EVD outbreaks. (**A**) Model describing the spatio-temporal pattern (STP) of outbreaks between 2001 and 2014. (**B**) Model based on forest loss (FL) for the period 2006 and 2014. Yellow points indicate outbreak locations; red points represent random locations with no report of EVD outbreaks. Compared to random locations, favorability based on FL is significantly higher for seven of the nine outbreaks that occurred after 2005. The map of Africa shows the area represented by axes x and y; country borders are outlined in grey; the green area represents rainforests (GlobCover version 2.1 database for 2005–2006, © ESA/ESA Globcover 2005 Project, led by MEDIAS-France/POSTEL).

The spatio-temporal (STP) model detected a significant (χ^2^ = 55.286; *p* = 2.74 × 10^−8^) spatio-temporal trend in which 16 of the 17 EVD outbreaks during 2001–2005 were clustered around the Gabon-Republic of Congo border, in the western Congo basin. After 2006, 8 of the 9 outbreaks occurred in Uganda and eastern/southern Democratic Republic of Congo (DRC), and after 2013 in West Africa (Fig. 1A, Tables S3 and S4).

**Figure 2:**
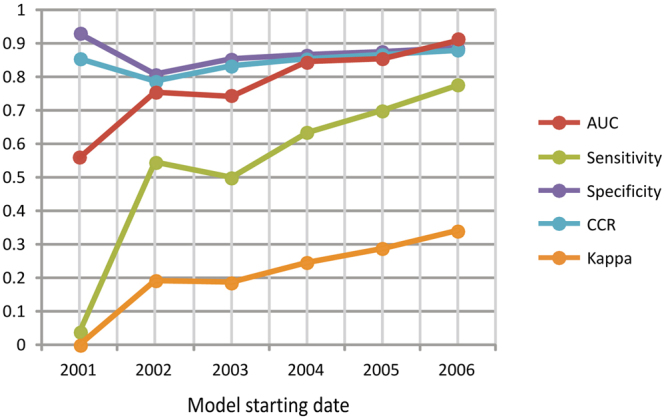
Capacity of discrimination (Area Under the Curve, AUC) and classification [sensitivity, specificity, Correct Classification Rate (CCR) and Kappa] of the six models defining the favorability of the occurrence of EVD outbreaks due to forest loss (FL). The x-axis is the starting year of the time period considered in the models.

Importantly, the 6 forest loss (FL) models only selected variables indicating forest changes less than 3 years prior to the outbreaks (Table S3). In all cases, higher proportions of dense forest losses significantly favored later EVD outbreaks. The most significant FL model (χ^2^ = 25.925; *p* = 0.000033), and with the highest capacity to discriminate between presences and absences of EVD outbreaks (AUC = 0.910), was that generated for the period 2006–2014 (Fig. 2, Table S4). This model indicated favorable conditions (F > 0.5) for 7 of the 9 EVD outbreaks in this period —those in Uganda, DRC and Guinea, all close to the limits of the rainforest biome (Fig. 1B) — and low favorability (F < 0.5) for 89% of non-outbreak locations. The variable most significantly associated with outbreaks, according to this model, was the proportion of ‘not intact forest with dense cover (>83%) and tall trees (>19 m)’ lost in the buffer area the same year as the outbreak occurred (Wald = 7.421; p = 0.006) (Figures S2 and S3, Table S3). The densest canopy cover (>83%) characterized 3 out of the 4 variables in the model. However, both intact and previously disturbed forests, and a wide range of tree heights (5–>19 m) were represented in the final model.

The FL models that were run considering periods 2003–2014, 2004–2014 and 2005–2014 were sensitive to the 7 outbreaks explained by the 2006–2014 model, but they did not provide any evidence for a relation between forest loss and outbreaks prior to 2006. Only the FL model for 2002–2014 (χ^2^ = 20.7; p = 0.000114) described favorable conditions for outbreaks that occurred before that year, although with less overall discrimination (AUC = 0.755) and classification power than the 2006–2014 model (Fig. 2, Table S4). Because of this, both 2002–2014 and 2006–2014 time periods were further considered for variation partitioning analyses (See Supplementary Methods).

**Figure 3:**
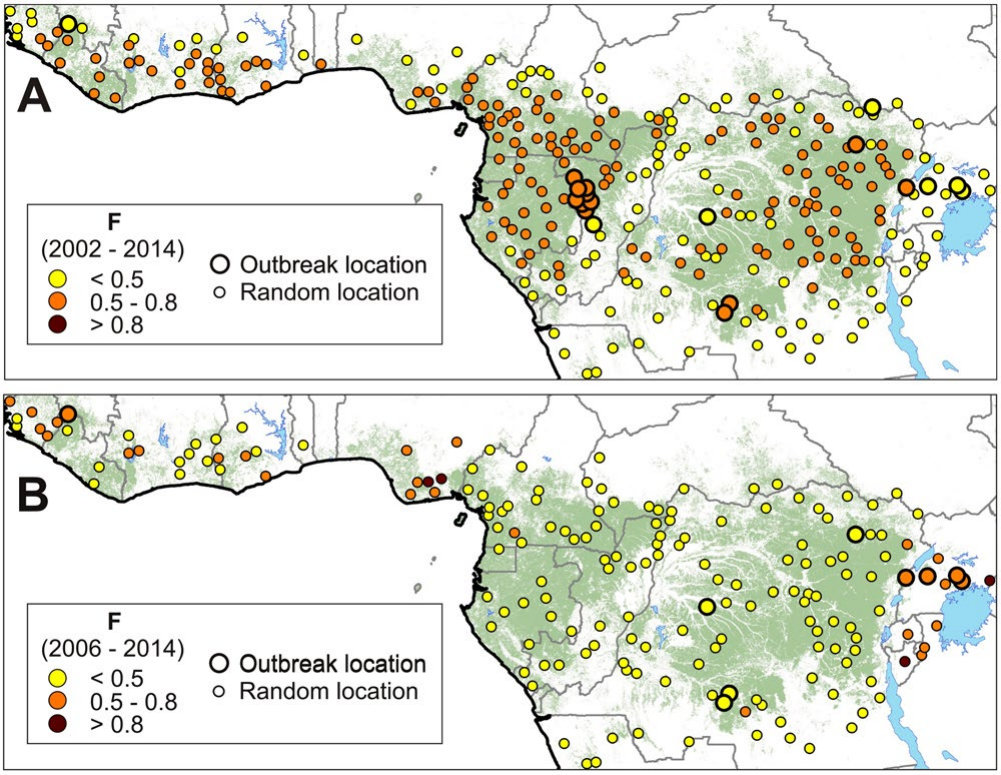
Models based on the basal spatial favorability (BSF) for EVD. Point colors indicate favorability (ranging 0–1). (**A**) The explanatory variable in the model for the period 2002–2014 is the environmental/zoogeographical favorability for the occurrence of Ebola virus in the wild (12). (**B**) The explanatory variable in the model for the period 2006–2014 is the rural human population density. Maps were generated using ArcGIS 10.3 (http://desktop.arcgis.com/en/).

Significant basal spatial favorability (BSF) models were only found for the periods 2001–2014, 2002–2014 and 2006–2014. In the two former periods, there was a significant association (χ^2^ > 5.000; *p* < 0.025) between favorability for the Ebola virus and outbreaks. This supports that differences in environmental potential for Ebola virus presence in the environment were related to the probability of occurrence of EVD outbreaks (Fig. 3, Table S3). However, this does not apply to the period 2006–2014, when outbreaks —which occurred along the limits of the West and Central African rainforest distribution range— were moderately associated to denser human populations (χ^2^ = 2.771; *p* = 0.096). Nevertheless, the discrimination and classification capacities of the BSF model between 2006 and 2014 were not as good (AUC = 0.812; sensitivity = 0.556; kappa = 0.175) as those based on forest loss (AUC = 0.910; sensitivity = 0.778; kappa = 0.341) (Table S4).

**Figure 4:**
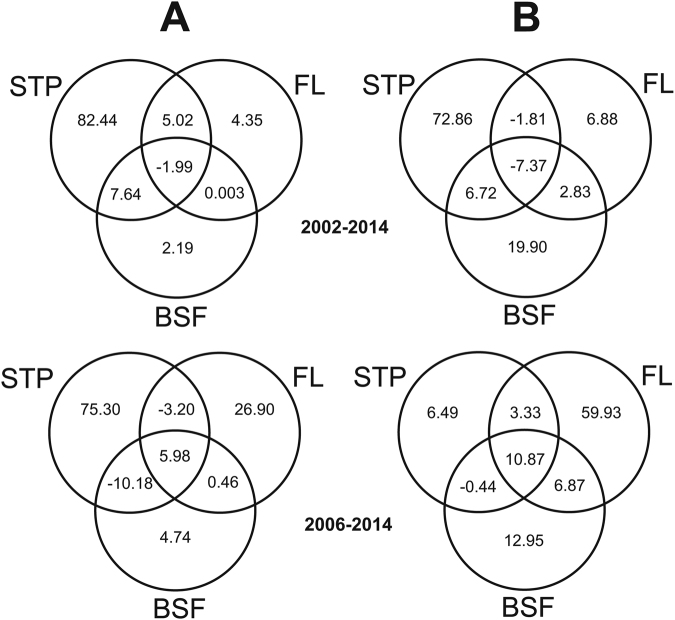
Venn diagrams displaying the results of variation partitioning analyses of a model combining spatio-temporal pattern (STP), forest loss (FL) and basal spatial favorability (BSF) for EVD outbreaks, for the periods 2002–2014 and 2006–2014. (**A**) Analysis focused on the complete Ebola-virus area (EVA)^18^. (**B**) Analysis focused on the range of favorability values overlapping with EVD outbreaks (EVDA).

Variation partitioning analyses regarding the complete Ebola virus area^18^ (EVA) revealed that, in the period 2002–2014, the contribution made by forest loss (FL) alone to explain the variation in favorability for EVD outbreak was low (4.35%) compared with that made by the spatio-temporal pattern (STP) (82.44%) (Fig. 4). However, the importance of forest loss increased dramatically to 26.90% for the period 2006–2014. In the area within the range of favorability values overlapping with EVD outbreaks (EVDA), forest loss alone accounted for 6.88% of the variation (compared with 72.86% for the STP) in 2002–2014, but increased to 59.93% (vs. only 6.49% for the STP) in 2006–2014. From 2006 to 2014, the basal spatial favorability (BSF) explained a low proportion of the EVD outbreaks in EVA (4.74%), while gaining relevance in EVDA (12.95%), but even then, forest loss alone was 4.6 times more relevant (59.93%) than the contribution of BSF alone.

Our study independently supports recent findings of an association between EVD outbreak locations and forest loss after 2004^16^. In addition, the spatio-temporal approach and the inclusion of all outbreaks later than 2000 revealed that: (1) the EVD outbreak-forest loss link is only significant around the limits of the West and Central African rainforest biome and excludes other EVD areas in the western Congo basin; (2) there is a time lag between forest loss and EVD outbreaks of 2 years; and (3) the loss of dense forests, principally those with >83% canopy cover, is an important factor. We also show that zoonotic EVD outbreaks appear in areas where human population density is high and where the virus has favorable conditions, but the relative importance of forest loss is partially (>60%) independent of these factors (Fig. 4).

The coupling between EVD outbreaks and forest loss in the margins of the rainforest biome within the previous two years, highlighted in our study, has profound implications. A plausible explanation is that contact between humans and infected wildlife increases dramatically after the removal of forest. Such an effect has been previously suggested^22,23^, and while our results strongly support such an interpretation, they also indicate that the changes are not sustained beyond two years. A variety of ecological descriptors (e.g. species richness) are affected soon after forest fragmentation^24^, and the factors promoting the emergence of the Ebola virus (host range, reservoir species, circulation in nature) are still unknown^5,21^. Forest loss disrupts animal movements and local densities, and thus influences their interactions and the potential for a pathogen to be transmitted between individuals and across species —though for Ebola such mechanisms remain theoretical. Regardless of whether or not fruit bats are important reservoirs of Ebola virus^6^, these animals are evidently involved in the virus’ ecology^25,26^. Deforestation influences fruit bat movement and abundance^23,27,28^, and the composition, abundance and behaviors of the wider mammal fauna is influenced by timber cutting and disturbance^29^. Thus, forest loss and fragmentation could favor the combination of ecological events that are required for viral emergence. Interestingly, our results, which are not limited to tall intact old growth forests, highlight the association between EVD outbreaks and close-canopy forests.

EVD outbreaks may increase in the coming decades. Human population growth, and greater penetration into Africa’s remaining forests will be coupled with the proliferation of potential reservoir species as urbanization, agriculture and deforestation intensify^30–32^. Greater population mobility via improved roads and air access increases the risks of an undetected EVD outbreak becoming a pandemic. A rapid response to any outbreak is fundamental to reducing contagion and reducing mortality, but increased preparation and vigilance are needed to diminish the risk. The challenge, however, is enormous. The vast and still relatively inaccessible areas involved, alongside the limited resources and manpower available, make it clear that priority setting will better guide monitoring and ensure preparedness. Health services in frontier areas require bolstering but the need for interdisciplinary approaches to improve our understanding of the ecology of the virus and its hosts, as we suggest in our study, cannot be neglected^30^. Our results provide indicators that could be useful for predicting where and when EVD outbreaks are more likely to occur. The data availability and image processing requirements of rapid predictions appear well within current technical abilities, though the best way to update forest cover classifications, or measures, requires further evaluation (to balance feasibility and utility). Such an approach would consider inhabited areas around the limits of the West and Central African rainforest biome favorable for Ebola, and identify where dense cover forests has been lost in the previous two years. This predictive system would draw special attention to high-risk locations, which can be updated and improved as data and concepts advance. For example, our analyses do not reveal why EVD outbreaks prior to 2005 occurred deep in the rainforest biome —within Republic of Congo and Gabon— but subsequently shifted to more transitional forests —in Uganda, DRC and Guinea. These regional shifts could result from inter-annual variation in rainfall across the continent^33,34^ and may reflect fruit availability and associated movements though more data would be needed to clarify these putative relationships.

Prevention of EVD outbreaks is the ultimate goal. Accepting the inferred links to land cover as causal implies that the risk of zoonotic EVD outbreaks can be diminished by (1) reducing deforestation and (2) reducing human proximity and access to recently damaged forests (for two years). More generally, our results show that forest loss, like EVD, should be seen as a major global health issue and should be managed and funded accordingly.

## Methods

### Vegetation Cover

We selected 27 locations where Ebola virus disease (EVD) outbreak “index cases” (i.e. also primary cases or patients zero, the initial identified patients in specific outbreaks assumed to result from zoonotic transmission) had occurred (Table S1), and 280 locations without outbreaks as control areas (see criteria for buffer selection below in the section “Deforestation”). We evaluated vegetation-cover changes within a buffer area of radius 20-km around each location selected. Based on past work in West and Central Africa we judged 20 km the distance that could typically be traveled by hunters^20,21^. Within these buffers we described vegetation cover changes as a set of dependent variables used in our models (Table S2). The use of non-overlapping buffers prevented the possible problems arising from spatial autocorrelation among the data.

Forest loss was estimated from data of vegetation cover changes contained in the University of Maryland’s Global Forest Change (GFC) project^17^. The GFC dataset is a time-series analysis of Landsat images characterizing forest extent and change at a 30-meter spatial resolution. This dataset reports annual forest extent, loss, and gain from the period 2001–2014. Because trees are simply defined as vegetation taller than 5-m in height and with a canopy cover of more than 25%, losses of natural forest or planted vegetation are not distinguishable using GFC data. Given this constraint, we applied the forest cover classification developed by Tyukavina *et al*.^35^, who stratified forests according to 7 distinct classes, based on canopy structure, as defined by percent cover, height, and intactness according to Potapov’s *et al*.^36^ description of intact forest landscapes (IFL, defined as an unbroken expanse of natural ecosystems within the zone of current forest extent, showing no signs of significant human activity, and large enough that all native biodiversity, including viable populations of wide-ranging species, could be maintained)^36^:

1. Forest with low cover (between 25–45% canopy cover).
2. Forest with medium cover and short trees (45–83% canopy cover, 5 to 11-m height).
3. Forest with medium cover and tall trees (between 45–83% canopy cover, ≥11-m height).
4. Not IFL with dense cover and short trees (>83% canopy cover, <19-m height).
5. IFL with dense cover and short trees (with no signs of human disturbance, >83% canopy cover, <19-m height).
6. Not IFL with dense cover and tall trees (>83% canopy cover, ≥19-m height).
7. IFL with dense cover and tall trees (pristine old-growth natural forests, >83% canopy cover, ≥19-m height).

Forest area loss within each 20-km radius buffer area was estimated annually for the period 2001–2014. This estimation was made in ha (i.e. absolute forest loss, AFL), and also in percentage respect to year 2000 (i.e. relative forest loss, RFL). In order to characterize the type of forest to which quantified tree-losses were related, we made all calculations with reference to these forest strata:

1. Total forest (sum of strata 1 to 7).
2. Dense forest (sum of strata 4 to 7).
3. IFL (sum of strata 5 and 7).
4. Every forest stratum (1 to 7) separately.

We developed a second set of variables to describe patterns of forest fragmentation (Table S2), again employing data from the Global Forest Change (GFC) project^17^:

1. Mean distance to forest edge (MDFE)^24^: To quantify the grade of forest fragmentation, we calculated the average distance between every forested pixel within each 20-km radius buffer area and its nearest non-forest pixel. This variable was conceived as a spatial, not temporal characteristic of landscape, and so the calculations were referred to a single time period, for which we selected year 2001 (i.e. the date of the first EVD outbreak considered).
2. Increased edge (IE)^24^: This variable describes the change in relative length of the limit between forest and non-forest. We followed three steps: (1) calculate the length of forest edge in 2000 (i.e. the year before the study period) and in 2014 (i.e. the end of the study period); (2) calculate the increase in length between 2000 and 2014 (i.e. length in 2014 minus length in 2000); (3) calculate the proportion of increase with respect to the length in 2000.

MDFE and IE were calculated for total forest, for dense forest and for IFL.

### Deforestation

Our dataset included the locations and dates of 28 EVD outbreak “index cases” since 2001 (Table S1). Two outbreaks in Ekata (Gabon) occurred in the same year and were merged for our analysis; thus, operatively, the number of outbreak locations considered was 27.

Deforestation data were available from 2001 to 2014. Thus, given that our working hypothesis was that EVD outbreaks might occur as a consequence of deforestation, only cases within this period were considered.

In the search for ecologically meaningful relationships between deforestation and outbreaks, we needed to analyze events within both spatial and temporal contexts. We examined the geographic overlap of deforestation and EVD outbreaks and the time-scale involved. Six time lags after deforestation events were examined: 0 to 5 years. As the outbreaks considered in the study included all index cases between 2001 and 2014, it was not possible to examine time lags higher than 0 in the outbreaks of 2001, time lags higher than 1 in those of 2001 and 2002, and so on until 2006, for which time lags from 0 to 5 years were available in the data set. Because of this, the associations between outbreaks and forest loss were investigated by means of six temporally nested analyses, comprising from 9 to 14 years, all of them ending in 2014 (Table S5).

For our comparisons we selected 280 control locations where outbreaks have never occurred (Figure S1). All these locations were assigned random coordinates in three axes: latitude, longitude and time, considering the period 2001 and 2014. The geographical context was West and Central Africa.

Locations where EVD outbreaks have occurred, as well as the 280 randomly selected non-outbreak control locations were assessed for forest cover change. The random selection of non-outbreak locations was, however, subject to the following constraints:

1. All such locations were favorable for Ebola virus in wildlife^18^.
2. A human population was present, according to at least one of the following sources:

- Global Rural-Urban Mapping Project, Version 1 (GRUMPv1), Settlement Points (Palisades, NY: NASA Socioeconomic Data and Applications Center SEDAC) http://dx.doi.org/10.7927/H4M906KR.
- Interactive Forest Atlas of Cameroon, CAR, Congo, DRC, Equatorial Guinea and Gabon, http://www.wri.org/our-work/project/congo-basin-forests.
- Country, Cities and Places GIS Shapefile Map Layers, http://www.mapcruzin.com/.
- The Humanitarian Data Exchange (HDX) https://data.hdx.rwlabs.org/, NGIS Country Files http://geonames.nga.mil/gns/html/namefiles.html.

Because all our EVD index cases were in rural areas, we restricted our selection of control sites to such context. For this aim, we excluded human populations less than 20-km far from urban areas as the MODIS 500-m Map of Global Urban Extent defines them.

3. These locations were more than 40 km far from each other and from EVD outbreak locations, so that 20-km radius buffers did not overlap.

The final set of locations in the analysis consisted of 27 circles around EVD outbreak sites, and another 280 similar areas corresponding to non-outbreak sites.

### Modelling

The Favorability Function (F, whose range is 0–1) can be defined by both equations 1 and 2 ^19,37^:

1 $$F=\frac{\frac{P}{(1-P)}}{\frac{{n}_{1}}{{n}_{0}}+\frac{P}{(1-P)}}$$

where *P* is the probability of occurrence of an EVD outbreak according to the set of predictor variables included in the model; *n*_1_ is the number of EVD outbreaks considered in the model; *n*_0_ is the number of randomly selected non-outbreak control locations considered in the model;

2 $$F=\frac{{e}^{y}}{\frac{{n}_{1}}{{n}_{0}}+{e}^{y}}$$

where *e* is the base of natural logarithms; *y* is a linear combination of predictor variables defining *P*. Both *P* and *y* were calculated with logistic regression, using IBM-SPSS Statistics 23. To avoid the inclusion of redundant variables in a model, and with some exception mentioned below, a forward stepwise procedure was performed according to the significance of Rao’s^38^ score test and the Akaike Information Criterion (AIC)^39^. This procedure identifies the most significant and informative simple model (with only one predictor) and then nests it in increasingly more complex models while guaranteeing that a variable was added only if it had an additional significant contribution to the previous information and that the new more complex model was more efficient that the previous one. The parameters in the models were fitted by iteratively maximizing the log-likelihood of the model. We used χ^2^ tests to assess the model’s goodness of fit, and Wald tests to assess the contribution of every predictor variable in the model.

By considering different sets of predictors (Table S2), we modeled:

1. The spatio-temporal pattern (STP) of EVD outbreak occurrences. EVD outbreaks could possess some geographical and temporal association (i.e. autocorrelation), depending on factors beyond the scope of this study (e.g. macroclimatic phenomena)^33,40^ which could drive to a clumped distribution^41,42^. We analyzed the STP in order to avoid errors in inference that can arise from this autocorrelation. We used the Favorability Function to build a STP model describing the distribution of EVD outbreaks according to a set of variables resulting from combinations of latitude, longitude and the year of the outbreak (Table S2). We developed this method based on the trend surface approach^41^, but added time as a third dimension so that the model finally define a “trend volume” describing the 3D-pattern of EVD outbreak occurrences in Africa between 2001 and 2014. Variables in the STP model were recruited by backward stepwise selection; this enabled a better description of the spatio-temporal pattern by allowing model entry of a higher number of variables than with a forward stepwise approach.
2. The spatio-temporal association between forest loss (FL) and EVD outbreaks. We run a set of FL models to define environmental favorability for the occurrence of EVD outbreaks in humans, according to changes happening in the forest immediately before the outbreak, and according to the fragmentation patterns observed. We applied the Favorability Function according to the set of forest-change variables described above (see also Table S2). In order to minimize Type-I errors arising from the large number of variables employed, we used Benjamini & Hochberg’s^43^ False Discovery Rate (FDR) procedure. Since six temporally nested FL models were produced, considering a different set of possible time lags between forest loss and EVD outbreaks (see above), model comparisons were made according to their discrimination (area under the ROC curve or AUC)^44^ and classification capacities (sensitivity, specificity, correct classification rate and Kappa)^45^; the model with the most successful assessment was selected.
3. The association of other environmental factors (i.e. “basal spatial favorability” or BSF) and EVD outbreaks. Even supposing that forest loss were involved in the occurrence of EVD outbreaks, index cases of EVD in humans could be subject to two other basic requirements: (1) suitable environmental conditions for the pathogen to proliferate, and (2) a sufficient density of people to permit infection and contagion. Olivero *et al*.^18^ addressed the mapping of favorable areas for the Ebola virus in the wild as a function of climate (i.e. annual temperature range), *terra-firme* rain forest and some specific mammal types of distribution in Africa. We built a set of BSF models by applying the Favorability Function to two predictor variables: (1) favorability for the occurrence of Ebola virus in the wild according to Olivero *et al*.^18^; and (2) rural human population density as defined by a combination of the LandScan™ 2008 High Resolution Global Population Data Set and the MODIS 500-m Map of Global Urban Extent. Six temporally nested BSF models were defined and compared with the same criteria as in the FL models (see above).

Finally, we estimated the relative contribution of each STP, FL and BSF in explaining where and when EVD outbreaks occur, by integrating their predictor variables into a single favorability model. We then used variation partitioning analysis^46^, following the approach described by Muñoz *et al*.^47^ (see “Variation Partitioning Analysis” in Supplementary Materials). Variation partitioning was applied twice separately: (1) considering the whole range of favorability values (0–1), representing the complete Ebola-virus area (EVA) as defined by Olivero *et al*.^18^; and (2) considering the area within the range of favorability values overlapping with EVD outbreaks (EVDA).

## Electronic supplementary material

Supplementary Materials
