# Supplementary material for: Recent loss of closed forests is associated with Ebola virus disease outbreaks
Source: Sci Rep. 2017 Oct 30;7:14291. doi: 10.1038/s41598-017-14727-9 (PMC5662765; doi:10.1038/s41598-017-14727-9)
Supplement: Supplementary file 1 — Supplementary Materials [file 41598_2017_14727_MOESM1_ESM.pdf]

Supplementary Materials for

Recent loss of closed forests is associated with Ebola virus disease outbreaks

Jesus Olivero*, Julia E. Fa*, Raimundo Real, Ana L. Marquez, Miguel A. Farfán, J. Mario Vargas, David Gaveau, Mohammad A. Salim, Douglas Park, Jamison Suter, Shona King, Siv Aina Leendertz, Douglas Sheil and Robert Nasi

*Correspondence to: Email: jesusolivero@uma.es; jfa949@gmail.com

This file includes:

Variation Partitioning Methods

Supplementary Figures S1-S3

Supplementary Tables S1-S5

Variation Partitioning Analysis

The partial contribution of the factors defining the STP, FL and BSF models in explaining the occurrence of EVD outbreaks was analyzed by integrating their predictor variables into a single favorability model (hereafter named combined favorability, CF). We then used a variation partitioning analysis[1], following the approach described by Muñoz et al.[2]. Details of this method are given below:

Let's "pure effect" be defined as the contribution of a factor to explaining CF that is not influenced by the covariation of the other factors. Then, "shared effect" could be defined as the extent to which the proportion of CF that is explained by a factor cannot be distinguished from the proportion explained by another factor (i.e. the intersection of both factors' contributions). Finally, "apparent effect" is defined as the total contribution of a factor, considering both its pure and shared effects on CF.

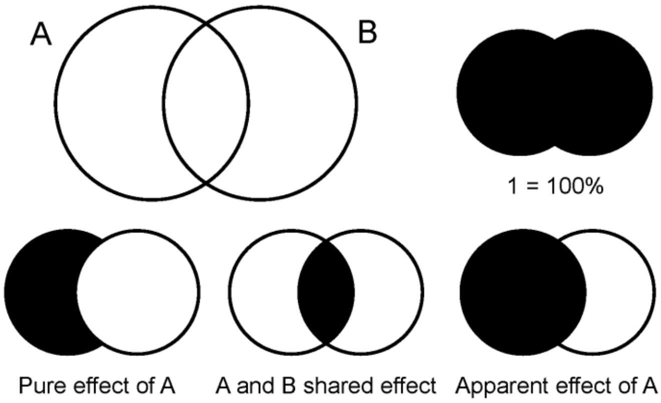

We determined which part of the CF variation was accounted for by the pure effect of each individual factor, and which proportion was accounted by the shared effect of more than one factor. Putting the focus on the STP factor, the apparent effect of STP was estimated using the square of Spearman's correlation coefficient (R^2) between CF and STP: R^2_{STP}; similarly, we calculated R^2_{FL} and R^2_{BSF}. The apparent effect of two factors together was estimated using the square of Spearman's correlation coefficient between the CF and a favorability model combining these two factors. For example, the apparent effect of STP and FL together was estimated by R^2 between the CF and a model combining both STP and FL: R^2_{STP+FL}; similarly, we calculated R^2_{FL+BSF} and $R^2_{STP+BSF}$.

Then, the pure effect of every factor was assessed by subtracting the apparent effect of the two other factors together from 1 (i.e. from the 100% of the CF variation):

$$\text{STP pure effect} = P_{STP} = 1 - R^2_{FL+BSF}$$
$$\text{FL pure effect} = P_{FL} = 1 - R^2_{STP+BSF}$$
$$\text{BSF pure effect} = P_{BSF} = 1 - R^2_{STP+FL}$$

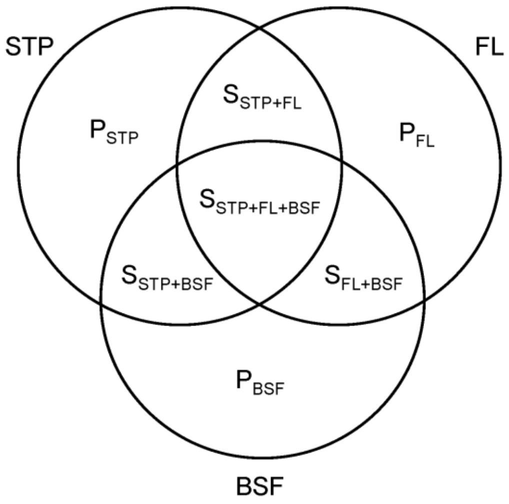

The effect shared by two factors was assessed with the following equations:

$$\text{STP and FL shared effect} = S_{STP+FL} = R^2_{STP+FL} - P_{STP} - P_{FL} + P_{BSF} - R^2_{BSF}$$
$$\text{STP and BSF shared effect} = S_{STP+BSF} = R^2_{STP+BSF} - P_{STP} - P_{BSF} + P_{FL} - R^2_{FL}$$
$$\text{FL and BSF shared effect} = S_{FL+BSF} = R^2_{FL+BSF} - P_{FL} - P_{BSF} + P_{STP} - R^2_{STP}$$

The effect shared by the three factors was assessed with the following equation:

$$S_{STP+FL+BSF} = 1 - (P_{STP} + P_{FL} + P_{BSF} + S_{STP+FL} + S_{STP+BSF} + S_{FL+BSF})$$

References

1. Borcard et al. Partialling out the spatial component of ecological variation. *Ecology* **73**, 1045-1055 (1992).
2. Muñoz, A.R. et al. Modelling the distribution of Bonelli's eagle in Spain: implications for conservation planning. *Divers. Distrib.* **11**, 477-486 (2005).

Supplementary Fig. S1 A. Distribution, in space and time, of EVD outbreaks between 2001 and 2014. **B.** Outbreak cases (yellow points) represented together with 280 randomly selected points with no outbreaks recorded (blue points). Maps were generated using ArcGIS 10.3 (http://desktop.arcgis.com/en/).

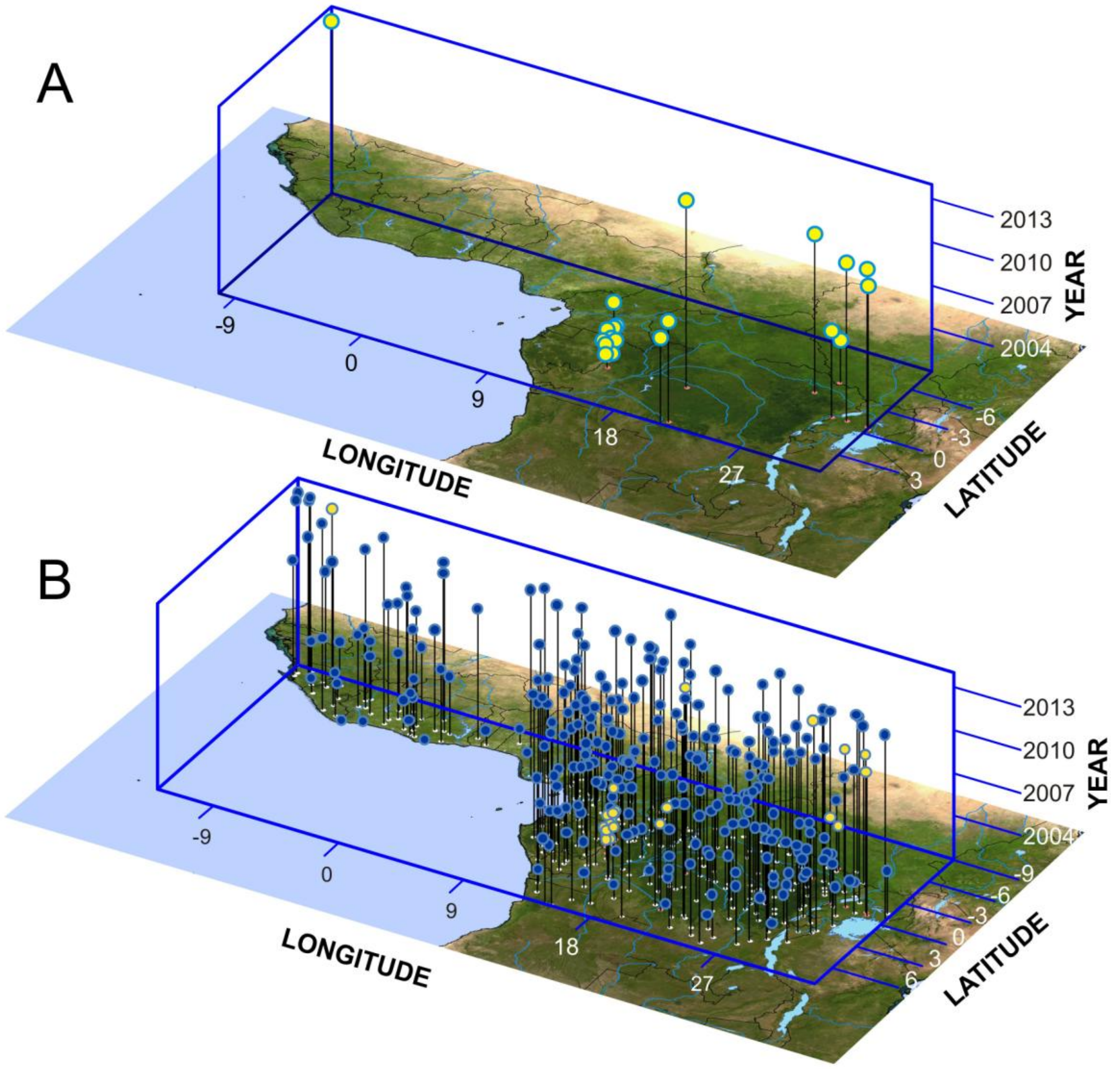

Supplementary Fig. S2. Favorability for the occurrence of EVD outbreaks plotted against the four variables in the model focused on years 2006-2014. Red dots indicate outbreak locations. 1: Luebo (DRC, 2007); 2: Budinbugyo (Uganda, 2007); 3: Mweka (DRC, 2008); 4: Nakisamata (Uganda, 2011); 5: Kibaale (Uganda, 2012); 6: Isiro (DRC, 2012); 7: Luwero (Uganda, 2012), 8: Guéckédou (Guinea, 2013); 9: Isaka-Ikanamongo (DRC, 2014) (see Fig. S3).

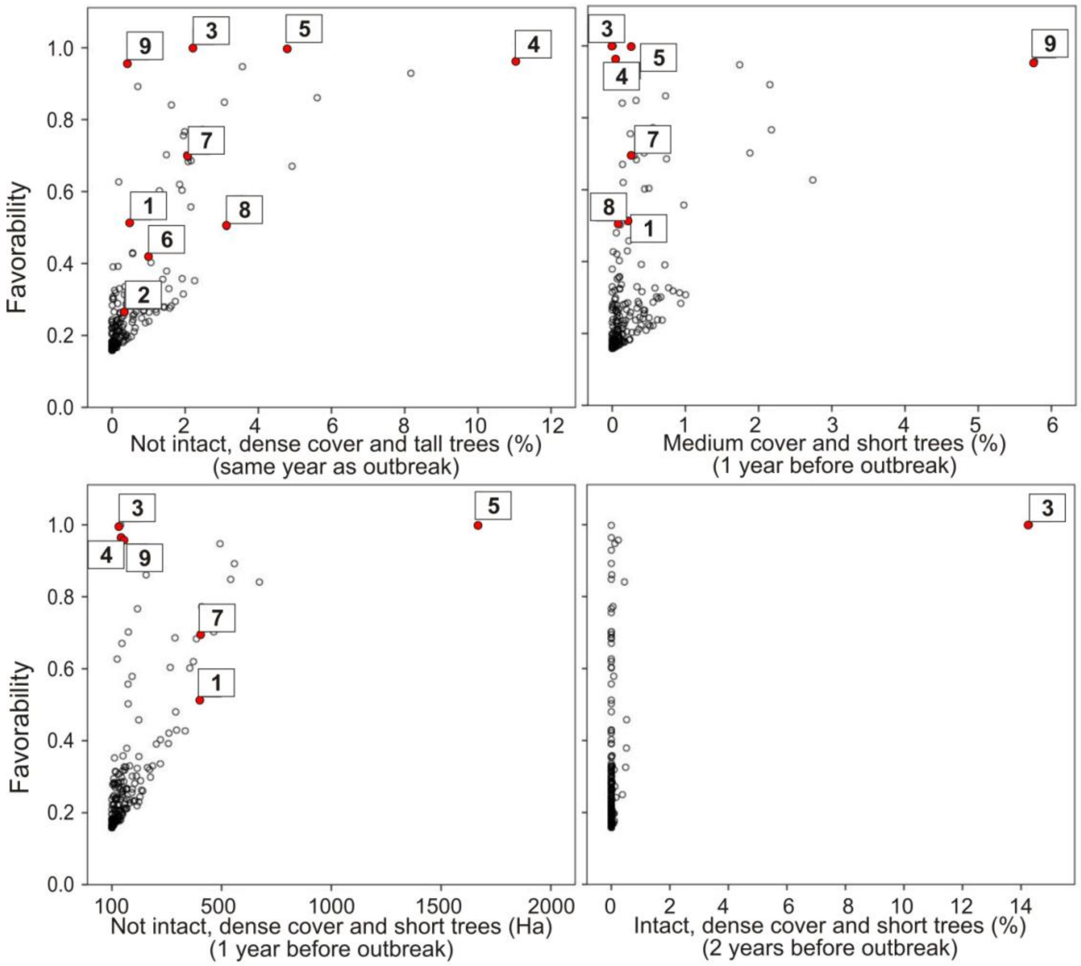

Supplementary Fig. S3. Locations, in central and west Africa, of the seven EVD outbreaks between 2006 and 2014 that have been significantly related to events of forest loss. Rectangles show the 20-km buffer areas around the outbreak locations, and the deforested surface since 2001. Type of forest and year of losses with significant relevance for the corresponding outbreaks are indicated. Maps were generated using ArcGIS 10.3 (http://desktop.arcgis.com/en/).

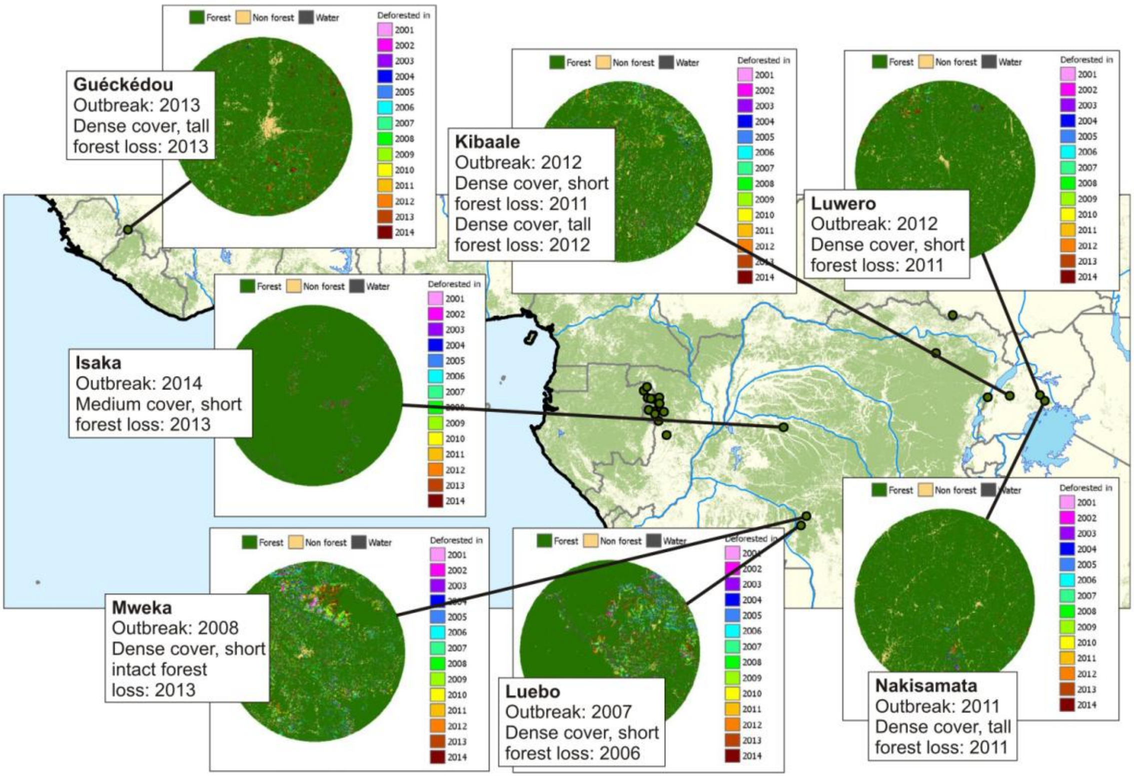

Supplementary Table S1. Referenced EVD outbreaks in humans. List and bibliographic references of localities where the occurrence of EVD outbreaks (events of zoonotic transmission of EVD to humans) has been confirmed. DRC: Democratic Republic of the Congo; CAR: Central African Republic; ROC: Republic of Congo. The two outbreaks in Ekata were merged for analysis purposes, as they occurred in the same year.

**Year**	**Country**	**Locality name**	**Reference***
2001	Gabon	Mendemba (Kikwit)	1, 2, 3, 4
2001	Gabon	Ekata	1
2001	Gabon	Ekata	1
2001	Gabon	Etakangaye	1, 2
2001	ROC	Olloba	1, 2
2002	ROC	Abolo	1
2002	ROC	Ambomi	1
2002	ROC	Entsiami	1, 2, 3
2002	Gabon	Grand Etoumbi	1, 2, 3
2002	ROC	Olloba	3, 5
2002	ROC	Kéllé	6, 7
2002	ROC	Mbomo	6, 7
2002	ROC	Yembelengoye	1, 2
2003	ROC	Mvoula	1, 2
2003	ROC	Mbanza	1, 2, 3, 5, 8, 9, 10
2003	ROC	Mbomo	5, 11
2004	Sudan	Yambio	1, 12, 13
2005	ROC	Etoumbi	3, 8
2007	DRC	Luebo	14, 15
2007	Uganda	Budinbugyo	16, 17
2008	DRC	Mweka	18, 19
2011	Uganda	Nakisamata	20
2012	Uganda	Kibaale	21
2012	DRC	Isiro	21
2012	Uganda	Luwero	21
2013	Guinea	Guéckédou	22, 23
2014	DRC	Isaka (Ikanamongo)	15, 24

***References:**

1. X. Pourrut *et al. Microb. and Infect.* **7**, 1005-1014 (2005).
2. E.M. Leroy *et al. Science* **303**, 387-390 (2004).
3. S.A. Lahm *et al. Trans. R. Soc. Trop. Med. Hyg.* **101**, 64-78 (2007).
4. World Health Organization (WHO) *Wkly. Epidemiol. Rec.* **26**, 223-228 (2003).
5. P. Rouquet *et al. Emerg. Infect. Diseases* **11**, 283-290 (2005).
6. P. Formenty *et al. Med. Trop. (Mars)* **63**, 291-295 (2003).
7. World Health Organization (WHO) *Wkly. Epidemiol. Rec.* **33**, 285-296 (2003).
8. D. Nkoghe *et al. Trans. R. Soc. Trop. Med. Hyg.* **105**, 466-472 (2011).
9. International Federation of Red Cross and Red Crescent Societies, Republic of Congo: Ebola epidemic. *Information Bulletin* **2/03** (2003).
10. A. Lucht *et al. J. Infect. Dis.* **196**, S184-192 (2007).
11. X. Pourrut *et al.* BMC *Infect. Dis.* **9**, 159-169 (2009).
12. C.O. Onyango *et al. J. Infect. Dis.* **196**, 193-198 (2007).
13. World Health Organization (WHO). *Wkly. Epidemiol. Rec. 43*, 370-375 (2005).
14. E.M. Leroy *et al. Vector-Borne and Zoobiotic Dis.* **9**, 723-728 (2009).

15. A. Rosello *et al. eLIFE* **4**:e09015 (2015).
16. J.S. Towner *et al. PLoS Pathog.* **4**, 10.1371/journal.ppat.1000212 (2008).
17. A. MacNeil *et al. Emerg. Infect. Diseases* 16: 1969-1972 (2010).
18. Center for Disease Control and Prevention (CDC). U.S. Department of Health and Human Services (2009).
19. World Health Organization (WHO) http://www.who.int/csr/don/2009_02_17/en/ (accessed 24/11/2015) (2009).
20. T. Shoemaker *et al. Emerg. Infect. Diseases* **18**, 1480-1483 (2012).
21. C.G. Albariño *et al. Virology 442*, 97-100 (2013).
22. D.G. Bausch, L. Schward. *PLoS Negl. Trop. Dis.* **8**, 10.1371/journal.pntd.0003056 (2014).
23. S. Baize *et al. N. Engl. J. Med.* doi: 10.1056/NEJMoa1404505 (2014).
24. World Health Organization (WHO) http://www.who.int/csr/don/2014_08_27_ebola/en/ (accessed 22/10/2014) (2014).

Supplementary Table S2 Predictor variables used to make the models that define the spatio-temporal pattern (STP) of EVD outbreaks between 2001 and 2014, their association with forest loss and fragmentation (FL), and the basal spatial favorability (BSF) for the occurrence of EVD outbreaks.

**Variable abbreviation**	**Variable description**
**Spatio-temporal pattern (STP) model**	
La	Geographical latitude (degree) + 35*
Lo	Geographical longitude (degree) + 17*
T	Time (year)
La × Lo	
La × T	
Lo x T	
La^2	
Lo^2	
T^2	
La^2 × Lo	
La × Lo^2	
La^2 × T	
La × T^2	
Lo^2 × T	
Lo × T^2	
La × Lo × T	
**Forest loss and fragmentation (FL) models****	
AFL_1_0	Absolute forest loss (Ha); forest class 1; same year
AFL_1_1	Absolute forest loss (Ha); forest class 1; 1 year before
AFL_1_2	Absolute forest loss (Ha); forest class 1; 2 years before
AFL_1_3	Absolute forest loss (Ha); forest class 1; 3 years before
AFL_1_4	Absolute forest loss (Ha); forest class 1; 4 years before
AFL_1_5	Absolute forest loss (Ha); forest class 1; 5 years before
AFL_2_0	Absolute forest loss (Ha); forest class 2; same year
AFL_2_1	Absolute forest loss (Ha); forest class 2; 1 year before
AFL_2_2	Absolute forest loss (Ha); forest class 2; 2 years before
AFL_2_3	Absolute forest loss (Ha); forest class 2; 3 years before
AFL_2_4	Absolute forest loss (Ha); forest class 2; 4 years before
AFL_2_5	Absolute forest loss (Ha); forest class 2; 5 years before
AFL_3_0	Absolute forest loss (Ha); forest class 3; same year
AFL_3_1	Absolute forest loss (Ha); forest class 3; 1 year before
AFL_3_2	Absolute forest loss (Ha); forest class 3; 2 years before
AFL_3_3	Absolute forest loss (Ha); forest class 3; 3 years before
AFL_3_4	Absolute forest loss (Ha); forest class 3; 4 years before
AFL_3_5	Absolute forest loss (Ha); forest class 3; 5 years before
AFL_4_0	Absolute forest loss (Ha); forest class 4; same year
AFL_4_1	Absolute forest loss (Ha); forest class 4; 1 year before
AFL_4_2	Absolute forest loss (Ha); forest class 4; 2 years before
AFL_4_3	Absolute forest loss (Ha); forest class 4; 3 years before
AFL_4_4	Absolute forest loss (Ha); forest class 4; 4 years before
AFL_4_5	Absolute forest loss (Ha); forest class 4; 5 years before
AFL_5_0	Absolute forest loss (Ha); forest class 5; same year
AFL_5_1	Absolute forest loss (Ha); forest class 5; 1 year before
AFL_5_2	Absolute forest loss (Ha); forest class 5; 2 years before
AFL_5_3	Absolute forest loss (Ha); forest class 5; 3 years before
AFL_5_4	Absolute forest loss (Ha); forest class 5; 4 years before
AFL_5_5	Absolute forest loss (Ha); forest class 5; 5 years before
AFL_6_0	Absolute forest loss (Ha); forest class 6; same year
AFL_6_1	Absolute forest loss (Ha); forest class 6; 1 year before

AFL_6_2	Absolute forest loss (Ha); forest class 6; 2 years before
AFL_6_3	Absolute forest loss (Ha); forest class 6; 3 years before
AFL_6_4	Absolute forest loss (Ha); forest class 6; 4 years before
AFL_6_5	Absolute forest loss (Ha); forest class 6; 5 years before
AFL_7_0	Absolute forest loss (Ha); forest class 7; same year
AFL_7_1	Absolute forest loss (Ha); forest class 7; 1 year before
AFL_7_2	Absolute forest loss (Ha); forest class 7; 2 years before
AFL_7_3	Absolute forest loss (Ha); forest class 7; 3 years before
AFL_7_4	Absolute forest loss (Ha); forest class 7; 4 years before
AFL_7_5	Absolute forest loss (Ha); forest class 7; 5 years before
AFL_all_0	Absolute forest loss (Ha); all forest classes; same year
AFL_all_1	Absolute forest loss (Ha); all forest classes; 1 year before
AFL_all_2	Absolute forest loss (Ha); all forest classes; 2 years before
AFL_all_3	Absolute forest loss (Ha); all forest classes; 3 years before
AFL_all_4	Absolute forest loss (Ha); all forest classes; 4 years before
AFL_all_5	Absolute forest loss (Ha); all forest classes; 5 years before
AFL_dense_0	Absolute forest loss (Ha); forest classes 4, 5, 6 & 7; same year
AFL_dense_1	Absolute forest loss (Ha); forest classes 4, 5, 6 & 7; 1 year before
AFL_dense_2	Absolute forest loss (Ha); forest classes 4, 5, 6 & 7; 2 years before
AFL_dense_3	Absolute forest loss (Ha); forest classes 4, 5, 6 & 7; 3 years before
AFL_dense_4	Absolute forest loss (Ha); forest classes 4, 5, 6 & 7; 4 years before
AFL_dense_5	Absolute forest loss (Ha); forest classes 4, 5, 6 & 7; 5 years before
AFL_intact_0	Absolute forest loss (Ha); forest classes 5 & 7; same year
AFL_intact_1	Absolute forest loss (Ha); forest classes 5 & 7; 1 year before
AFL_intact_2	Absolute forest loss (Ha); forest classes 5 & 7; 2 years before
AFL_intact_3	Absolute forest loss (Ha); forest classes 5 & 7; 3 years before
AFL_intact_4	Absolute forest loss (Ha); forest classes 5 & 7; 4 years before
AFL_intact_5	Absolute forest loss (Ha); forest classes 5 & 7; 5 years before
RFL_1_0	Relative forest loss (%); forest class 1; same year
RFL_1_1	Relative forest loss (%); forest class 1; 1 year before
RFL_1_2	Relative forest loss (%); forest class 1; 2 years before
RFL_1_3	Relative forest loss (%); forest class 1; 3 years before
RFL_1_4	Relative forest loss (%); forest class 1; 4 years before
RFL_1_5	Relative forest loss (%); forest class 1; 5 years before
RFL_2_0	Relative forest loss (%); forest class 2; same year
RFL_2_1	Relative forest loss (%); forest class 2; 1 year before
RFL_2_2	Relative forest loss (%); forest class 2; 2 years before
RFL_2_3	Relative forest loss (%); forest class 2; 3 years before
RFL_2_4	Relative forest loss (%); forest class 2; 4 years before
RFL_2_5	Relative forest loss (%); forest class 2; 5 years before
RFL_3_0	Relative forest loss (%); forest class 3; same year
RFL_3_1	Relative forest loss (%); forest class 3; 1 year before
RFL_3_2	Relative forest loss (%); forest class 3; 2 years before
RFL_3_3	Relative forest loss (%); forest class 3; 3 years before
RFL_3_4	Relative forest loss (%); forest class 3; 4 years before
RFL_3_5	Relative forest loss (%); forest class 3; 5 years before
RFL_4_0	Relative forest loss (%); forest class 4; same year
RFL_4_1	Relative forest loss (%); forest class 4; 1 year before
RFL_4_2	Relative forest loss (%); forest class 4; 2 years before
RFL_4_3	Relative forest loss (%); forest class 4; 3 years before
RFL_4_4	Relative forest loss (%); forest class 4; 4 years before
RFL_4_5	Relative forest loss (%); forest class 4; 5 years before
RFL_5_0	Relative forest loss (%); forest class 5; same year
RFL_5_1	Relative forest loss (%); forest class 5; 1 year before
RFL_5_2	Relative forest loss (%); forest class 5; 2 years before
RFL_5_3	Relative forest loss (%); forest class 5; 3 years before
RFL_5_4	Relative forest loss (%); forest class 5; 4 years before
RFL_5_5	Relative forest loss (%); forest class 5; 5 years before

RFL_6_0	Relative forest loss (%); forest class 6; same year
RFL_6_1	Relative forest loss (%); forest class 6; 1 year before
RFL_6_2	Relative forest loss (%); forest class 6; 2 years before
RFL_6_3	Relative forest loss (%); forest class 6; 3 years before
RFL_6_4	Relative forest loss (%); forest class 6; 4 years before
RFL_6_5	Relative forest loss (%); forest class 6; 5 years before
RFL_7_0	Relative forest loss (%); forest class 7; same year
RFL_7_1	Relative forest loss (%); forest class 7; 1 year before
RFL_7_2	Relative forest loss (%); forest class 7; 2 years before
RFL_7_3	Relative forest loss (%); forest class 7; 3 years before
RFL_7_4	Relative forest loss (%); forest class 7; 4 years before
RFL_7_5	Relative forest loss (%); forest class 7; 5 years before
RFL_all_0	Relative forest loss (%); all forest classes; same year
RFL_all_1	Relative forest loss (%); all forest classes; 1 year before
RFL_all_2	Relative forest loss (%); all forest classes; 2 years before
RFL_all_3	Relative forest loss (%); all forest classes; 3 years before
RFL_all_4	Relative forest loss (%); all forest classes; 4 years before
RFL_all_5	Relative forest loss (%); all forest classes; 5 years before
RFL_dense_0	Relative forest loss (%); forest classes 4, 5, 6 & 7; same year
RFL_dense_1	Relative forest loss (%); forest classes 4, 5, 6 & 7; 1 year before
RFL_dense_2	Relative forest loss (%); forest classes 4, 5, 6 & 7; 2 years before
RFL_dense_3	Relative forest loss (%); forest classes 4, 5, 6 & 7; 3 years before
RFL_dense_4	Relative forest loss (%); forest classes 4, 5, 6 & 7; 4 years before
RFL_dense_5	Relative forest loss (%); forest classes 4, 5, 6 & 7; 5 years before
RFL_intact_0	Relative forest loss (%); forest classes 5 & 7; same year
RFL_intact_1	Relative forest loss (%); forest classes 5 & 7; 1 year before
RFL_intact_2	Relative forest loss (%); forest classes 5 & 7; 2 years before
RFL_intact_3	Relative forest loss (%); forest classes 5 & 7; 3 years before
RFL_intact_4	Relative forest loss (%); forest classes 5 & 7; 4 years before
RFL_intact_5	Relative forest loss (%); forest classes 5 & 7; 5 years before
MDFE_all	Mean distance to forest edge (km); all forest classes
MDFE_dense	Mean distance to forest edge (km); forest classes 4, 5, 6 & 7
MDFE_intact	Mean distance to forest edge (km); forest classes 5 & 7
IE_all	Increased edge (length of forest edge in 2014 / length of forest edge in 2000; all forest classes
IE_dense	Increased edge (length of forest edge in 2014 / length of forest edge in 2000; forest classes 4, 5, 6 & 7
IE_intact	Increased edge (length of forest edge in 2014 / length of forest edge in 2000; forest classes 5 & 7
**Basal spatial favorability (BSF) models**	
Fav_EV	Favorable areas for the Ebola virus in the wild as a function of climate, forest type and the types of distributions shown by mammals in Africa***
Rur_Pop_Den	Rural human population density****

* The summand is to turn latitude and longitude into positive values

** 1. Forest with **low cover** (between 25-45% canopy cover).
2. Forest with **medium cover** and **short trees** (45-83% canopy cover, 5 to 11-m height).
3. Forest with **medium cover** and **tall trees** (between 45-83% canopy cover, ≥11-m height).
4. Not intact forest (IFL) with **dense cover** and **short trees** (>83% canopy cover, <19-m height).
5. Intact forest (IFL) with **dense cover** and **short trees** (with no signs of human disturbance, >83% canopy cover, <19-m height).
6. Not intact forest with **dense cover** and **tall trees** (>83% canopy cover, ≥19-m height).
7. Intact forest with **dense cover** and **tall trees** (pristine old-growth natural forests, >83% canopy cover, ≥19-m height).

*** J. Olivero et al., Mammal Rev. **47**, 24-37 (2017).

**** Defined by a combination of the LandScan™ 2008 High Resolution Global Population Data Set and the MODIS 500-m Map of Global Urban Extent

Supplementary Table S3 Explanatory variables forming part of the models that define the spatio-temporal pattern of EVD outbreaks between 2001 and 2014 (STP), the association of EVD outbreaks with forest loss and fragmentation (FL), and the basal spatial favorability (BSF) for the occurrence of EVD outbreaks. Variable coefficients refer to the linear combination *y* in the Favorability Function (see equation 2). Wald's parameters are shown for comparisons of variable importance in the model. Significant BSF models were not found for the periods 2003-2014, 2004-2014 and 2005-2014. Variable abbreviations as in Supplementary Table 2.

Variable abbreviation	Coefficient	Wald
**Spatio-temporal pattern (STP) model** (χ^2 = 55.286; p = 2.74×10^{-8})		
La	3.870	3.835
Lo	2.565	2.884
T	-0.910	9.452
La × Lo	12.223	3.707
La^2	-18.917	2.916
Lo^2	-3.082	8.531
La × Lo^2	0.003	2.461
La^2 × T	0.009	2.892
Lo^2 × T	0.002	8.384
La × Lo × T	-0.006	3.831
Constant	1775.646	9.124
**Forest loss and fragmentation (FL) models**		
**2001 – 2014** (χ^2 = 4.109; p = 0.043)		
RFL_intact_0	1.017	1.954
Constant	-2.431	134.211
**2002 – 2014** (χ^2 = 20.7; p = 0.000114)		
RFL_3_1	0.787	13.405
RFL_intact_0	0.924	2.715
RFL_6_0	0.260	4.123
Constant	-3.363	90.719
**2003 – 2014** (χ^2 = 17.704; p = 0.001)		
RFL_6_0	0.329	5.308
RFL_5_2	0.561	0.375
AFL_4_1	0.002	2.908
Constant	-3.518	82.071
**2004 – 2014** (χ^2 = 20.700; p = 0.000121)		
RFL_6_0	0.376	6.400
RFL_5_2	0.579	0.367
AFL_4_1	0.003	3.395
Constant	-3.928	63.708
**2005 – 2014** (χ^2 = 21.243; p = 0.000094)		
RFL_6_0	0.380	6.328
AFL_4_1	0.003	3.660
RFL_5_2	0.597	0.345
Constant	-4.076	55.968
**2006 – 2014** (χ^2 = 25.925; p = 0.000033)		
AFL_4_1	0.003	3.409
RFL_2_1	0.741	4.828
RFL_5_2	0.594	0.532
RFL_6_0	0.432	7.421
Constant	-4.652	43.846
**Basal spatial favorability (BSF) models**		
**2001 – 2014** (χ^2 = 7.894; p = 0.005)		
Fav_EV	3.411	5.783
Constant	-5.021	18.140

**2002 – 2014** (χ^2 = 5.069; p = 0.024)		
Fav_EV	2.805	3.972
Constant	-4.614	16.052
**2006 – 2014** (χ^2 = 2.771; p = 0.096)		
Rur_Pop_Den	.006	3.546
Constant	-3.306	62.268

Supplementary Table S4. Assessment of the model that define the spatio-temporal pattern (STP) of EVD outbreaks between 2001 and 2014, their association with forest loss and fragmentation (FL), and the basal spatial favorability (BSF) for the occurrence of EVD outbreaks. AUC (Area Under the ROC Curve) evaluates the model discrimination capacity between occurrences and absences*. Sensitivity, specificity, CCR (Correct Classification Rate) and Kappa evaluate the model classification capacity (sensitivity is focused on occurrences, specificity is focused on absences, and CCR and Kappa are focused on both occurrences and absences)**.

**Years**	**AUC**	**Sensitivity**	**Specificity**	**CCR**	**Kappa**
**Spatio-temporal pattern (STP) model**					
**2001 - 2014**	0.879	0.808	0.736	0.742	0.247
**Forest loss and fragmentation (FL) models**					
**2001 - 2014**	0.559	0.039	0.932	0.856	-0.033
**2002 - 2014**	0.755	0.546	0.808	0.788	0.193
**2003 - 2014**	0.743	0.500	0.854	0.834	0.186
**2004 - 2014**	0.846	0.636	0.866	0.855	0.247
**2005 - 2014**	0.856	0.700	0.877	0.868	0.289
**2006 - 2014**	0.910	0.778	0.887	0.882	0.341
**Basal spatial favorability (BSF) models**					
**2002 - 2014**	0.656	0.682	0.433	0.452	0.029
**2006 - 2014**	0.812	0.556	0.842	0.828	0.175

***** J.M. Lobo et al., Global Ecol. Biogeogr. **17**, 145-151 (2008).
****** A.H. Fielding, J.F. Bell, Environ. Conserv. **24**, 38-49 (1997).

Supplementary Table S5. Time lags considered since a deforestation event to an EVD outbreak in humans, and correspondences with the period of years that can be considered and the number of outbreaks that can be included in a model.

Time lag (years)	Years considered	Number of outbreaks
0 - 5	2006 - 2014	9
0 - 4	2005 - 2014	10
0 - 3	2004 - 2014	11
0 - 2	2003 - 2014	14
0 - 1	2002 - 2014	22
0	2001 - 2014	26
